# Molecular diagnosis of severe bacterial sepsis in children

**DOI:** 10.1186/cc11754

**Published:** 2012-11-14

**Authors:** M Merisescu, M Luminos, G Jugulete, D Florea, A Streinu-Cercel

**Affiliations:** 1 National Institute of Infectious Diseases, Bucharest, Romania

## Background

Bacterial sepsis is a very severe infection, potentially lethal, that requires an etiologic diagnosis and fairly complex treatment, quickly set. Plex-ID is the newest method in etiologic diagnoses of bacterial infections. It can detect bacterial DNA found in various pathological products: blood, CSF, joint fluid, pleural liquid.

## Methods

We conducted a 6-month study from January to June 2012 on children admitted to our pediatric ICU of the National Institute of Infectious Diseases for severe forms of bacterial sepsis. In these children we monitored the following parameters: sex, age and origin (nosocomial or community-acquired infection). Positive diagnosis of sepsis was established on clinical and laboratory criteria (hemocultures, CSF cultures). As well as classical cultures, we found causal agents through the newest modern technique - Plex-ID. We watched for correlation of data obtained by classical methods versus molecular methods and the clinical and biological evolution of patients under treatment

## Results

In the 6 months of study, 21 children met the clinical and biological criteria for bacterial sepsis. Seventy-six percent of patients came from other hospitals, the reason for which we consider that the etiology is most probably nosocomial pathogens. Sex distribution was approximately equal boys and girls, and in terms of distribution by age group prevailed in children aged 0 to 3 years (80%). We obtained 15 positive results (71%) by molecular methods that were correlated with conventional methods of diagnosis. The seat in the distribution of case etiology was as follows: *Niessiera meningitides *(four cases), *Streptococcus pneumoniae *(two cases), *Staphylococcus *spp. (four cases), *Klebsiella pneumoniae *(one case), *Acinetobacter baumannii *(one case), *Pseudomonas aeruginosa *(one case), *Propionibacterium acnes *(one case), and *Haemophilus influenzae *(one case).

## Conclusion

Bacterial sepsis in children is a serious condition resulting in four deaths (19%) with septic shock and multiple organ failure in our study. The patients require rapid etiologic diagnosis and establishment of appropriate emergency treatment. Plex-ID is an effective and rapid diagnosis method, identifying casual agent in 71% of cases.

The major advantage of this new method is the possibility to establish the etiological diagnosis in the early hours of patient admission. Also the speed and specificity of the modern diagnosis methods increase the survival index of the hospitalized patients with severe bacterial sepsis.

